# Impact, Vulnerabilities, and Mitigation Strategies for Cyber-Secure Critical Infrastructure

**DOI:** 10.3390/s23084060

**Published:** 2023-04-17

**Authors:** Hugo Riggs, Shahid Tufail, Imtiaz Parvez, Mohd Tariq, Mohammed Aquib Khan, Asham Amir, Kedari Vineetha Vuda, Arif I. Sarwat

**Affiliations:** 1 Department of Electrical and Computer Engineering, Florida International University, Miami, FL 33174, USA; hrigg002@fiu.edu (H.R.); stufa001@fiu.edu (S.T.); mkhan139@fiu.edu (M.A.K.); aamir007@fiu.edu (A.A.); kvuda001@fiu.edu (K.V.V.); 2 Department of Computer Science, Utah Valley University, Orem, UT 84058, USA; imtiaz.parvez@uvu.edu

**Keywords:** computer networks, cyber attack, signal detection, machine learning, smart grid

## Abstract

Several critical infrastructures are integrating information technology into their operations, and as a result, the cyber attack surface extends over a broad range of these infrastructures. Cyber attacks have been a serious problem for industries since the early 2000s, causing significant interruptions to their ability to produce goods or offer services to their clients. The thriving cybercrime economy encompasses money laundering, black markets, and attacks on cyber-physical systems that result in service disruptions. Furthermore, extensive data breaches have compromised the personally identifiable information of millions of people. This paper aims to summarize some of the major cyber attacks that have occurred in the past 20 years against critical infrastructures. These data are gathered in order to analyze the types of cyber attacks, their consequences, vulnerabilities, as well as the victims and attackers. Cybersecurity standards and tools are tabulated in this paper in order to address this issue. This paper also provides an estimate of the number of major cyber attacks that will occur on critical infrastructure in the future. This estimate predicts a significant increase in such incidents worldwide over the next five years. Based on the study’s findings, it is estimated that over the next 5 years, 1100 major cyber attacks will occur on critical infrastructures worldwide, each causing more than USD 1 million in damages.

## 1. Introduction

With many examples of cyber attacks affecting critical infrastructure (CI) in recent years, it has become evident that these incidents are a major threat to the existing critical infrastructure and, thus, society as a whole [1,2,3,4,5,6,7,8,9,10]. In this paper, we define CI based on the definition from the Cyber & Infrastructure Security Agency. Critical infrastructure includes cyber and physical assets, systems, and networks of chemical and commercial facilities, communications, critical manufacturing, dams, defense industrial base, emergency services, energy, financial, food and agriculture, government facilities, healthcare, and public health, information technology (IT), nuclear reactors, materials and waste, transportation systems, and water and waste management. Not only are these sectors highly significant to modern countries but they also have strong interdependencies. A disruptive effect on one CI sector can have a cascading failure effect on other CIs, specifically, outages in electrical CIs affect most other CIs [11,12,13]. CIs are sets of physical and virtual assets, systems, and networks that provide a nation with economic security, public health, and safety [12]. This review focuses on electrical grid CIs due to the dependence that other CIs have on the electrical grid. The electrical grid has numerous remote terminal units for controlling physical systems and is a balanced system between load and generation. Cyber attacks against the electrical grid can lead to a cascading failure affecting most other CIs.

CI was listed in [13] as agriculture and food, water, public health and safety, emergency services, government, defense industrial base, information and telecommunications, energy, transportation, banking and finance, industry/manufacturing, postal, and shipping. CI is not immune to cyber attacks; for example, parts of the power grid in Ukraine have been taken down by malware named BlackEnergy3. Other examples show that industry-scale food processing plants have closed due to ransomware, and multiple businesses and their retail functions have been shuttered by malware embedded in trusted third-party updating services [14]. In March 2019, a denial of service (DoS) attack was launched against part of the Supervisory Control and Data Acquisition (SCADA) infrastructures of electric utilities in Utah, which shut down some of their observation capabilities. The exploitative programs that are in use today on the internet have enabled cybercriminals to acquire and update malicious programs with ease. The avenue for attack, once opened, allows the attacker to explore further attacks over potential vulnerabilities in the victim’s system. Communications in a network are often assumed to be private; however, an attacker may exploit the network in a man-in-the-middle attack to steal confidential information, sabotage a cyber-physical system, or maliciously alter information. Cyber attacks have malicious intents; they were identified in [15] as obstruction of information, undermining cybersecurity measures, retardation of the decision-making process, denial in providing public services, abatement of public confidence, lowering the reputation of the victim country, and destroying a legal interest.

### 1.1. Evolution of Cyber Attacks

Cybercrime has existed since the early days of computer networks, with ransomware attacks seen as early as 1989. The digitization of control systems in CI, which previously operated from electromechanical systems, embeds the vulnerabilities of the digital system. The opening for cyber attackers grows as CIs have evolved their operational technologies [16]. More advanced malware has been developed over the past three decades, posing a constant threat to CIs. Many types of malware are being developed by professional software development organizations and purchased by cyber attackers. This division of malware development and deployment depends on the growing cybercrime economy [16]. Over time, the complexity of malware has increased, and it is used for the ransom of computer systems and CI system sabotage. A modern attacker can source customized malware tools from third-party providers.

Ransomware is expected to be more commonly experienced in CI through the Internet of Things (IoT) and CPS. While the technical specifics of a cyber attack can vary, the general flow of such attacks follows a trend. The trend in industrial control system (ICS) cyber attacks involves initiating a phishing attack to obtain access or insider access to facility computers. With access, a download or local pen drive can deliver spying and control malware. This malware carries out the primary sabotage actions, and then the exfiltration of the computer system is done, often preceded by a kill disk operation. The kill disk operation writes a binary zero value for all bits in the computer system storage, temporarily rendering it useless [16].

An emerging type of attack is a false data injection attack (FDIA) that targets the data stream of state estimation measurement outputs to cause the system operator to take incorrect control actions, which can have a detrimental physical and economic impact on the power system. The FDIA depends on three assumptions. The first is that the attacker has experience with power system operations and the capabilities of the targeted system. Secondly, the attacker is capable of manipulating meter measurements. Thirdly, the attacker has knowledge of the network topology, system electrical parameters, an understanding of the SCADA system, and existing cybersecurity mechanisms [1,8]. Furthermore, as the power grid digitizes and transitions to a smart grid, implementing neural networks for prediction has been shown to be highly sensitive to even small manipulations of data [7]. A set of case studies involving FDIA attacks against voltage and current sensors of power converters in a photovoltaic (PV)-based microgrid concluded that malfunction due to FDIA in these sensors can damage the PV modules.

The falsified signal was removed from the control process using sensor malfunction detection and ride-through operations [17].

Furthermore, the FDIA vulnerability of the power grid being researched is automatic generator control vulnerability. Such an attack affects the frequency of the power grid by interrupting the ability of the load control center to calculate control values. This could be very damaging and potentially cause a blackout [2]. In a specific use-case presented in [9], considering distributed energy resources, FDIA on PV production meter data used in 15-min ahead forecasting is simulated and studied. The FDIA causes disruption in the control center communication with distributed energy resources (DER) assets simulated on an IEEE 34 bus system with three PVs, one synchronous generator, and one energy storage. The results showed that the FDIA can potentially lead to cascading failures by creating an overcurrent and voltage collapse.

### 1.2. Contributions in This Paper

This paper has the following key contributions: (a) provides a comprehensive set of major cyber attack categories for a holistic understanding of the threats and damages that can be expected from a cyber attack; (b) identifies standards and organizations that address cybersecurity in IT; (c) summarizes some of the historical major cyber attacks against critical infrastructure; and (d) identifies strategies and tools that cybersecurity teams will use as they build their defenses through both passive and active methods. The paper provides a description of seven major categories of cyber attacks, presents a 20-year history of significant (more than USD 1 million in damages) cyber attacks, and summarizes the way the systems were compromised.

### 1.3. Organization of the Paper

This paper focuses on the primary driving factors in cyber attacks and the types of cyber attacks. It also enumerates methods that are used by cyber security teams to counter these threats. The rest of the paper is structured as follows. Section 3 discusses the cybercrime economy and the types of cyber attacks on the CI. Section 4 provides a systematic process for building cyber defenses, it also discusses attribution techniques and the role of attribution; Section 5 covers existing standards that detail the frameworks and best practices that address cyber attacks; the section also shows a process for developing cyber-secure infrastructure. In Section 6 a discussion of the findings of the review is conducted. Finally, our conclusions are presented in Section 7.

## 2. Methodology for Review

The aim of our paper is to review and understand the reported information on cybersecurity research and incidents targeted at critical infrastructures, with a focus on the energy critical infrastructure, in order to identify areas that require future research. Research questions were devised to motivate the review and evaluate the identified publications. The contents of the publications and reports in the review are contextualized with the adversary techniques matrix published by the MITRE organization.

### 2.1. Research Questions

To evaluate the existing works on the impacts on critical infrastructure from cyber-incidents, research questions were identified.

- Question 1: *What are the motivations of cyber attackers?* To understand the motivations of the adversary, we searched for publications that detailed the modern economic structure of cybercrime. The cybercrime economy has been growing parallelly with the internet, enabling a robust network of adversarial actors and advanced persistent threats (APTs). Answering this question provides insight into the degree of sophistication of the adversary and the level of development.
- Question 2: *What are the types of cyber attacks on critical infrastructures?* To understand the technical aspects of a cyber attack, this question aims to list and describe the cyber attacks that can be used against critical infrastructures. Answering this question will help develop knowledge of cyber attacks and the means and mediums through which they can occur.
- Question 3: *How does a cyber attack impact critical infrastructure and how does it affect citizens?* To identify how critical infrastructure is impacted by cyber attacks, this question aims to create an understanding of the expected impacts that a cyber attack will have on critical infrastructure and gain some understanding of the effect it will have on citizens who depend on critical infrastructure services.
- Question 4: *How many significant cyber attacks on critical infrastructure have occurred, and which critical infrastructures are targeted?* By analyzing the records of significant cyber attacks to account for all of the attacks on the various critical infrastructures, this provides a perspective on the trend in cyber attacks and which CIs can be targeted.
- Question 5: *What mitigation strategies are in use to mitigate the effects of a cyber attack?* Answering this question will inform security operators about the various solutions that exist to enhance infrastructure protection against cyber attacks. If the mitigation strategies do not address all cyber attack techniques, they could help to identify areas that require future research.

The research questions require a systematic approach to be answered in-depth and such an approach was taken in developing the answers to these questions.

**Figure 1:**
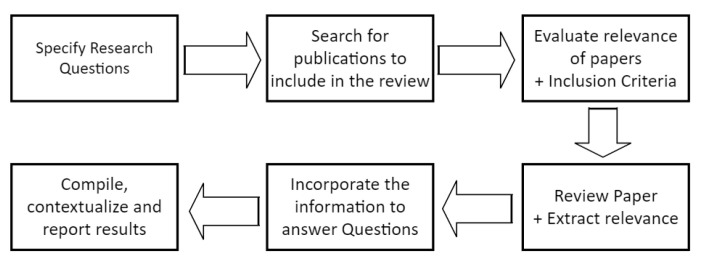
Method of approach to the review.

To answer these research questions a review and synthesis of the literature was conducted and followed the sequential process shown in Figure 1.

### 2.2. Selection of Papers and Reports

The selection criteria for identifying publications for inclusion in the review are as follows.

- Directly reports the events of a historical cyber attack.
- Directly implements a study of attack detection and prevention.
- Describes the organizational aspects of cybercrime.
- Describes the implementable network technology practices for mitigation of cyber attacks.

Peer-reviewed publications in international journals and conferences, along with theses, are all considered for review. The search for publications was primarily conducted through online databases, such as IEEE Xplore, ACM, Science Direct, and Springer Link. Additionally, white papers from high authority publishers and organizations in the cybersecurity industry were included, including the SANS Institute.

**Figure 2:**
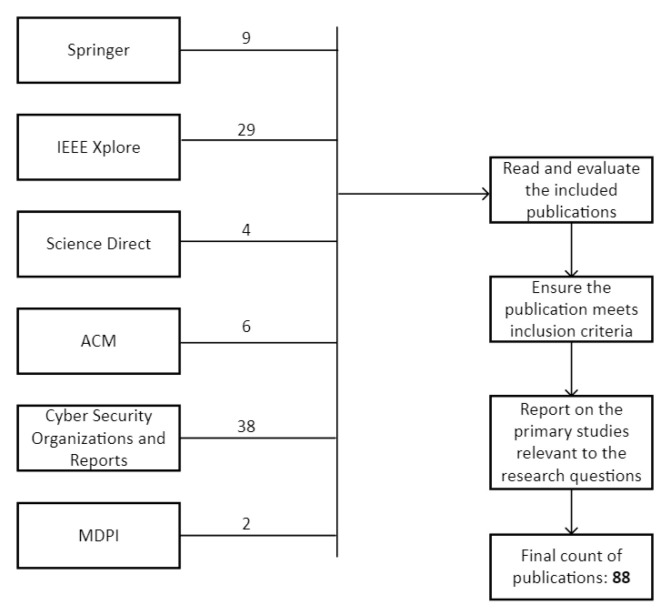
Publication selection and inclusion.

The sourcing of publications and articles along with the evaluation of the selected materials is done in a systematic way, shown in Figure 2. The various publishing entities, the numbers of selected works, and the process of evaluation, inclusion, and reporting on the selected works are listed.

## 3. Types of Cyber Attacks on Critical Infrastructure

**Figure 3:**
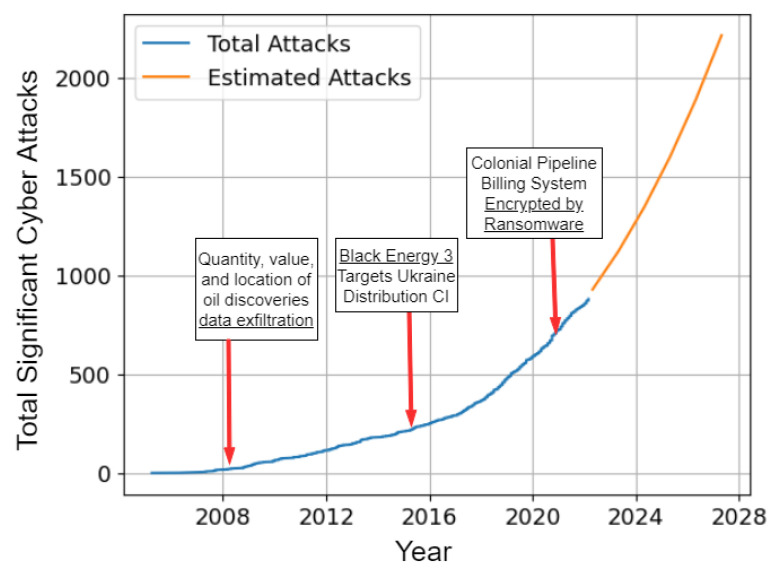
Estimate: cyber attacks will increase exponentially.

**Figure 4:**
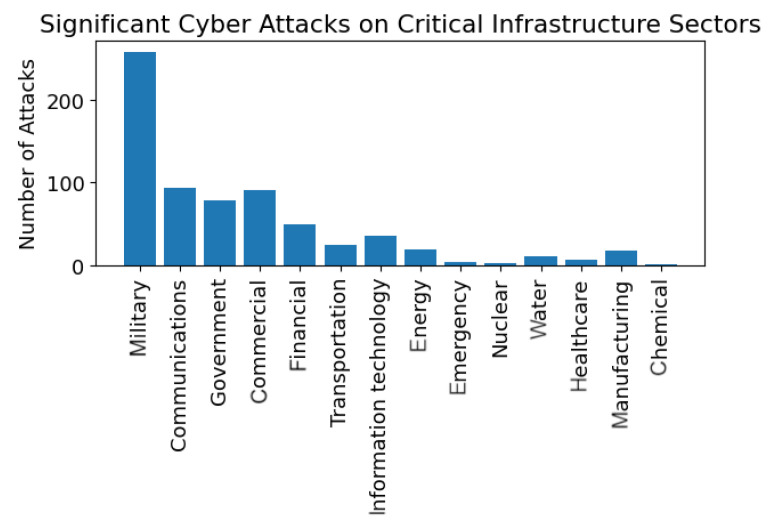
Significant cyber attacks by the CI sector since 2006, analyzed from the CSIS incidents list.

The projection shown in Figure 3 was conducted from the data collected by the Center for Strategic and International Studies (CSIS) in Washington, D.C. The CSIS provides a significant cyber attack list [18]. The CSIS defines a significant cyber attack as one that results in at least USD 1 million in damage. Significant cyber attacks are defined as cyber attacks on government agencies, defense, and high-tech companies, or attacks on other CIs that cause losses of more than USD 1 million Figure 3 shows the total number of significant cyber attacks measured and includes a projection of expected attacks through 2025. The projection, using polynomial regression, shows that there will be more significant cyber attacks in the next five years than the combined significant cyber attacks since 2005. The list from CSIS was further analyzed based on a keyword search to relate the cyber attack to a specific critical infrastructure. For example, if the cyber attack targeted a military base, it was attributed to the military CI, and if an attack contained the words financial or banking, it was included in the financial CI. The significant attacks per-CI are shown in Figure 4. The rest of this section expands on the discussion of the disruptive cybercrime economy. The section also enumerates the various top-level cyber attack types with some of their sub-variants.

### 3.1. Cybercrime Economy

The cybercriminal economy has emerged worldwide, enabling many types of cyber attack functions as a service. However, while the focus is on cyber attacks in these sections, the cybercrime economy enables many other types of criminal activity. In [5], a literature review yields an extensive and consistent survey of the services used by the cybercrime business, organized using the value chain perspective, to understand cyber attacks systematically. Further, an understanding of the specialization, commercialization, and cooperation in coordinating a cyber attack is developed. They identify 24 value-added activities and their relations in the cybercrime market. These can be offered “as a service” for use in a cyber attack. The framework in [5] of cyber attacks “as a service” helps us understand the modern cybercriminal ecosystem and hacking innovations. Some services that facilitate cyber attacks include training and recruiting, development of exploitative software, scanning networks, denying service, phishing, target ranking, and money laundering. These services are provided as subscriptions, licenses, pay-per-records, or commission-based services [5,19]. The prominent concern for CIs is APTs. APTs are groups that are supported by their host nations and perform long-term targeting of the victim’s CI. The general goal of APTs is to steal data from the victim. However, they also target the control management systems and components [19] of CI. The critical importance of the power infrastructure to the socioeconomic stability and the effect of blackouts make the smart grid a primary target [6]. APTs represent a subset of the cybercrime economy, and an APT is often a benefit to the host nation’s economy, as they are compensated for their actions. This is due to the subterfuge of critical infrastructures slowing the economies of competitors to the host nation. An emergent factor for the electrical infrastructure is electricity theft, which is a major contributor to nontechnical losses in the distribution systems of the smart grid [4].

#### Money Laundering, Theft, Black Markets, and Ransom

One role of the cybercrime economy is in money laundering. This activity is evident in the use of cryptocurrencies for financial exchange from the victims to the attacker. A cryptocurrency transaction occurs, such as a ransom payment, and it is exchanged into another currency by the attacker. Cryptocurrencies lend themselves to this practice as they are functional currencies for communication networks that operate outside of traditional banks [20]. Trojan malware can facilitate information theft. If an enterprise system is compromised and the database is accessed to steal personally identifiable information, this information can be sold online. Online black markets exist, and they are frequently pursued by law enforcement and shut down. However, popular and well-known digital black markets commonly re-emerge at a new location, as moving software frameworks throughout different IT infrastructures is easily facilitated [21,22,23]. Another example of the function of the cybercriminal economy involves the ransom of critical computer systems. These ransomware-based attacks are targeted against critical services, such as utilities and hospitals [24]. The reasons for targeting these services are clear. They are critical for the public, and victims are willing to pay significant amounts of money to free their computer systems from ransomware. This is simply because it is less expensive for them to pay the ransom and recover their systems than remain out of operation [25].

### 3.2. The Ransomware Cyber Attack

A ransomware’s malicious action is to either encrypt, lock, or exfiltrate data, and the ransomware will be specialized for the target platform. The variety of operating systems means that system-specific libraries and functions will be used by the ransomware to perform malicious actions. Mostly, they will target PC/workstations with a Windows operating system [16]. Within the cybercrime economy, some groups operate as Ransomware-as-a-Corporation (RAAC). Attackers operating as RAAC frequently issue press releases and use corporate language in their communications. If the ransom is not paid, then the victims’ operational systems will remain inaccessible, and any critical personal information that has been exfiltrated will be posted on a dark web leak site to damage the company’s reputation and business processes [21].

Although current ransomware campaigns do not target CPS, the installation of more intelligent electronic devices in the field by CI makes the CI and its CPS a more likely target for ransomware. As smart technologies continue to expand and integrate into homes, transportation, buildings, and throughout cities, they will become a growing target in the future development of ransomware that targets this new environment. Thus, ransomware that targets industrial CPS intelligent electronic devices will become more prevalent [16,26,27,28].

Most commonly, e-mails are the delivery method of ransomware. Malicious e-mails carry ransomware as an attachment, which contains the malware. These messages are often sent as spam broadcast to as many e-mail addresses as possible or can be directed and tailored to specific individuals or organizations. More details on targeted e-mailing are given in Section 3.5. The attachment can provide a link or file that initiates the installation of ransomware [16].

Encryption ransomware prevents victims from accessing their files by encrypting them with a secret key. The key and decryption software are then used for ransom. With advances in ransomware design, more targeted algorithms are used in encryption to specifically target file types of higher value to the victim. This reduces the time needed to perform the malicious encryption action after infecting the victim’s computer. Locking ransomware has a similar goal to encryption-based malware, but it targets locking mechanisms designed to lock a system, such as a master boot record lock, screen lock, or computer desktop lock. The malware uses built-in security systems to lock the victim out of their computer system [29]. Finally, an information theft ransomware exfiltrates personally identifiable information (PII) from a victim’s computer. The stolen PII is advertised to the victim as blackmail, and ransom is paid to prevent the publishing of the PII.

#### Supply Chain Ransomware

This type of ransomware is distributed through a trusted software distribution mechanism, particularly through a software updater provided by an IT service company. The attack was worldwide and affected businesses such as pharmacies, railways, and storefronts. The attack exploited a vulnerability in the IT service company’s software updating system, which compromised the businesses that relied on it for updates [30].

### 3.3. Denial of Service

In the DoS attack the attacker prevents the intended user from accessing a resource. The attacker can reduce the intended user’s access to the server by flooding the network i.e., increasing the traffic to disrupt access to a service. The attacker also attempts to break the connectivity between two systems [31]. Flooding services make the system receive too much traffic for the servers to handle. Flooding a system slows the system down and can ultimately halt the system.

The implication of DoS-based electricity theft against the energy CI is shown in the experimental results. The growing installation of intelligent electronic devices in CPS and the Internet of Things (IoT) domestic devices, such as connected homes and smart appliances, also increases the potential damage to CI from DoS attacks. The proliferation of more internet-connected grid technologies creates an increased vulnerability to such attacks [3].

#### 3.3.1. Flooding in Mesh Networks

A utility can implement advanced meter infrastructure (AMI) using large wireless mesh networks. However, delays in wireless sensor networks can be caused by network flooding attacks. A malicious node in a wireless mesh network can tamper with messages that are sensitive to flooding attacks, resulting in a saturation of the AMI network. The DoS attack will come from a malicious node or nodes in the mesh network, sending excessive unnecessary data packets throughout the network and issuing excessive requests for communication. This traffic congests the mesh network and forms the basis of the flooding attack, which is identified as a DoS and impacts the network by increasing the latency of the communications [32,33].

IEC 62351 assigns digital signatures as a requirement for low-latency critical communication in ICS. However, digitally signed messages in wireless mesh networks are vulnerable to flooding DoS attacks, as demonstrated in [34], in which a model of phasor measurement data collection and transmission was subjected to flooding DoS. The flooding blocked the phasor measurement unit from transmitting data to the load flow control center. This type of interruption can affect the decision-making processes of the control center and generation control centers. In [3], an experiment with a consumer meter was performed, in which the meter was subjected to a flooding cyber attack. The flooding attack caused the meter to under-report the average watt-hour consumed at a rate of 1.77% less reported power consumption after four days. Other intelligent electronic devices may also be targeted. In [35], experimental signal jamming is performed on wireless networks against IEC 62351-based technologies. The GOOSE substation protocol is evaluated on a WiFi-based wireless power network, and the reactive jamming resulted in an 88% degraded throughput. Time-critical messaging is affected, resulting in latency overshooting the maximum message delay constraints.

#### 3.3.2. Incidents of Denial of Service Attack

In 2000, a DoS attack on Yahoo rendered the site non-operational for more than 3 h. The attack was based on a Smurf attack and a Tribe Flood Network Technique. Through this attack, Yahoo received data requests of around or greater than one gigabyte per second [31]. Another DoS attack on the electric grid operations of Los Angeles County in California and Salt Lake County in Utah interrupted the electrical system operations for more than 10 h. It affected the computer systems used within the electrical utilities responsible for running the office functions. The attack had little impact on power delivery, but it raises concerns about the future if proper steps are not taken to mitigate such attacks [36].

### 3.4. Man-in-the-Middle

This Man-in-the-Middle (MITM) cyber attack is a kind of cyber attack where an outsider enters between two communication nodes and tries to remain undetected. The MITM can change the routed information before the information reaches the other node. This cyber attack accesses, reads, changes, or modifies the secret information without the victim’s detecting manipulation. One capability involves injecting new messages and another involves the capacity to intercept all messages. Despite cryptography, a successful MITM attacker compromises exchanges between two systems. The MITM is either a passive listener or imitates one of the parties and manipulates the data sent. There may be many objectives for an attack either using the data overheard for a subsequent action or changing the data before it reaches the other party. The attacker extracts information to be used in many ways: fraud, unapproved support exchanges, blackmail, credential theft, and spying [37].

A MITM attack intercepts the victim’s activity through the attacker’s system before it is routed to its intended destination. The attacker gains access to an unsecured network, often targeting networks in public areas such as Wi-Fi access points [37]. This provides the attacker with an avenue to deploy tools that intercept information between the victims, often targeting personal computers where their connection to websites is monitored. This can result in credentials, financial details, and personally identifiable information being captured [37]. There are several types of MITM attacks, and the man-in-the-browser variant injects malicious software into the victim’s computer or mobile device through phishing. Upon clicking on a phishing e-mail link or opening the attachment, the user loads the malware, and the malware installs itself on the browser without the user’s knowledge. The malware enables the attacker to capture the information between the victim and specific websites. Exploits that are used to enter a MITM include internet protocol (IP) spoofing, address resolution protocol (ARP) spoofing, global navigation satellite system (GNSS) spoofing, and domain name system (DNS) spoofing [38].

#### 3.4.1. IP Spoofing

In IP spoofing, the attacker modifies the source address in the IP packet header to make the receiver believe that the packet was received from a trusted site. From the victim’s side, the packets will be received as though they were sent from a trusted source. However, the IP source reported in the packet is modified and does not represent the actual source [39].

#### 3.4.2. ARP Spoofing

ARP spoofing involves sending a false ARP reply message to the default network gateway, claiming to associate the MAC address with the target’s IP address. This ARP protocol translates IP addresses to MAC addresses. MITM ARP packets transmit over LAN by sending malicious ARP packets to a default gateway on the local area network [40]. The re-association request from the attacker can enable them to appear as the default gateway for traffic; thus, all other hosts in the network will transmit their data through the MITM.

#### 3.4.3. DNS Spoofing

In DNS spoofing, the IP address in a DNS record is replaced by an IP address in the control of the attacker. This redirects internet traffic to fraudulent websites that resemble intended destinations [41,42,43].

#### 3.4.4. HTTPS/SSL Hijacking

Stolen data can be decrypted using several methods, including HTTPS spoofing, SSL hijacking, SSL stripping, and others. In HTTPS spoofing, the attacker uses a domain that appears identical to the target website’s domain. In SSL hijacking, the attacker passes the produced authentication keys to both the client and application during a TCP handshake [44]. This seems, by all accounts, to be a safe association when the MITM controls the whole session. In SSL stripping, the attacker sends a decoded form of the application’s site to the client by maintaining the anchored session with the application. Meanwhile, the client’s whole session is noticeable to the attacker.

### 3.5. Phishing and Remote Execution

Phishing and remote attacks rely on social engineering methods designed to have the victim reveal sensitive information or use malicious software. Phishing is highly prevalent in cyber attacks on CIs and it is identified in many of the significant cyber attacks in Table 1. Attackers send fraudulent communication to coerce a victim into sharing classified credentials or other information. Credentials obtained can be used to perform other attacks, such as the installation of malware, remote access, or the theft of information. Attackers may ransom credentials through the threat of publication [45,46,47].

Phishing methods are also used to introduce ransomware infections on the victim’s network infrastructure [48]. The 2020 Federal Bureau of Information’s Internet Crime Report lists phishing as the most common cyber attack performed against US citizens by a wide margin, likely due to the increasingly sophisticated methods that cybercriminals use. The report lists 241,342 complaints of phishing in 2020; the next highest reported crime was non-payment or non-delivery of goods through online transactions, with a total of 108,869 complaints [49].

**Figure 5:**
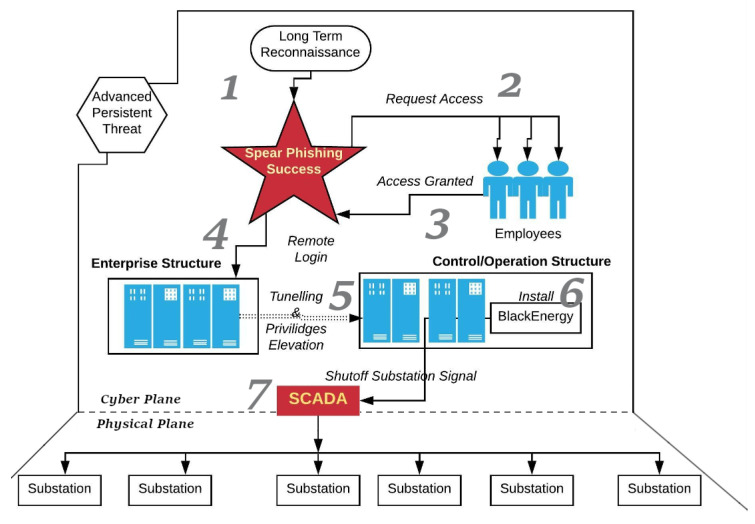
Targeting employees with socially engineered phishing campaigns, leading to remote sabotage.

Phishing was used to target the Ukrainian Power grid. In the lead-up to Christmas in 2015, attackers took full control of remote terminal units in the Ukrainian power distribution grid and used them to change the set points on breakers. This action triggered the opening of critical breakers, de-energizing around 225,000 customers for an extended duration [14]. Phishing was the initial means by which attackers gained access to perform remote connection sabotage. Furthermore, in December 2016, attackers disabled energy delivery from a Kiev transmission station by using phishing to initiate remote sabotage, which caused a one-hour outage [50]. The flow of the phishing and remote execution cyber attack against the energy distribution system CI is shown in Figure 5. The attack sequence is framed as a separation of the cyber and physical planes, highlighting the sequential process of the attack by the APT. The process starts with reconnaissance, followed by a phishing campaign, gaining access, tunneling into OT, installing malware in OT, and finally using human-machine interfaces to sabotage physical systems in the field. The flow is captured in Figure 5.

#### 3.5.1. Bulk Phishing

The most common form of phishing (bulk phishing) involves broadcasting messages through emails that are not personalized or targeted towards a specific individual or company. Attackers typically impersonate banking services, email/cloud providers, and streaming services to obtain credentials from potential victims.

#### 3.5.2. Spear Phishing

In contrast to bulk phishing, ‘spear phishing’ includes methods of attack intended to target a specific organization or person with tailored communication. To increase the chances of deceit, attackers gather and use personal information about their target. Spear phishing targeted Hilary Clinton’s 2016 presidential campaign by Threat Group-4127 [51].

#### 3.5.3. CEO Phishing and Whaling

Whaling and chief executive officer (CEO) fraud represent two specific types of spear phishing tactics. Whaling involves phishing targeting CEOs or senior executives. CEO fraud is a reciprocal tactic in which the phishing attempt is made to impersonate the CEO [52].

#### 3.5.4. Clone Phishing

Clone phishing is another phishing attack; in this tactic, attackers manipulate the link/attachment files included in an otherwise legitimate email. Using a previously delivered email, attackers will attempt to clone an email and include malicious attachments in place of original files and links. This form of phishing typically requires that one of the parties, either the sender or the recipient of the email, has previously had their account compromised [45].

#### 3.5.5. Additional Phishing Tactics

Phishing is practiced in attacks outside of email communication, as well. Voice phishing involves attackers spoofing a phone number to resemble a trusted institution. Attackers will dial large quantities of phone numbers and play automated recordings that try to coerce sensitive information to help resolve an issue on the victim’s account [53]. Finally, page hijacking is another form of phishing in which attackers will compromise or mimic legitimate web pages and redirect users to malware or an exploit kit utilizing cross-site scripting [54].

### 3.6. False Data Attack (Parameter/Command Injection)

False data injection is an attack that attempts to corrupt the control data. FDIA is presented in three types [7]:

- Targeted constrained FDIA: In this type of attack, data are injected after clear analysis, with a known amount of data inserted to appear realistic.
- Targeted unconstrained FDIA: In this type of attack, the attacker attempts to corrupt the values of some variables, and those variables in turn corrupt the remaining dependent variables.
- Random FDIA: In this type of attack, data packets are randomly distributed without consideration of the real values.

#### 3.6.1. Protocols without Encryption in the CI

Digitization and ubiquitous computing have found their way into areas once solely operated by electromechanical controls. False data injection in CI control systems of the energy sector can damage the power electronics hardware. Protocol-level challenges in securing cyber-physical systems within the energy distribution grid are apparent in Distributed Network Protocol 3 (DNP3), GOOSE, and Modbus, as these protocols transmit data without encryption [55]. These systems should operate on physically isolated networks. An additional method to enhance their security is through the use of *bump in the wire*, an encryption hardware that encrypts the transmitted data before they travel the wider network. A methodology for layer-by-layer analysis of protocols to identify vulnerabilities is provided in [55]. Understanding protocol-level weaknesses is key to a secure network. Cyber-physical systems that utilize data generated from sensors in their processing and interact with information are prime targets for FDIA attacks. The cyber-physical system uses sensor data to implement the network and control adjustments of power electronics. In certain cases, these systems also require low latency in communication, which can make encryption of communications impossible, such as in the IEC 61850 GOOSE standard. If voltage is incorrectly controlled, it can cause damage to the power electronics.

#### 3.6.2. Automatic Generator Control

FDIA on automatic generator control is a vulnerability that enables the manipulation of data in closed-loop control of generator control signals. This type of attack can cause significant damage to the generation and transmission equipment of the power grid, potentially leading to blackouts. This control system—if attacked by FDIA—will lead to overloading transmission lines by excessive power generation [56].

#### 3.6.3. Parameter Modification in Inverters

As the power grid becomes increasingly dependent on renewable energy sources, new grid services will emerge based on smart inverters (SI) connected to these sources. The settings of these smart inverters are critical for these grid services to operate optimally. The settings of these inverters represent a point where FDIA can be particularly damaging to the smart grid [10]. A SI attack can affect the SI functions for volt–var, volt–watt, and a constant power factor. Such attacks potentially impact voltage profiles, system losses, and the operation of voltage control legacy devices. In such cases of FDIA, the severity depends on the prevailing SI functions [10].

### 3.7. Worm and Trojan Malware

A computer worm is a computer virus that is characterized as a self-replicating malware that spreads across networks executing disruptive payloads [57]. A worm targets hosts by following these scan types:

- An active selective random scan or sequential scan, in which the worm scans for vulnerable hosts.
- A hit-list scan, where the worm creates a target list and then searches for susceptible hosts.
- A routable scan, which utilizes information about a network to select and scan the IP address space [57].

Using a routable IP address allows the worm to propagate quickly and effectively, avoiding some detection methods. Another characteristic of a worm is the target space or medium through which it propagates. This includes the internet, email, P2P, USB local, and more. The worm propagates either as self-carried or through a second channel. In the second channel method, the main malware payload is remotely downloaded by the base installer. The activation of a worm on a system uses a vulnerability in the host, and the worm may protect itself by modifying its binary code with encryption [58].

#### The Stuxnet Worm

Stuxnet is a computer worm that was initially found in Iran but has since spread worldwide. This worm targets the control systems of a nation’s critical infrastructure, and a successful attack by Stuxnet can result in the manipulation of the control system, causing disruption and damage to critical infrastructure and posing a threat to modern society. In 2010, Iran identified over 30,000 infected industrial computer systems, with Stuxnet specifically targeting nuclear power plant operational technology (OT) computers. The initial infections were at reactor core sites with flash memory used to introduce the worm locally. The worm targets an industrial control system that runs on Windows from Siemens [59].

### 3.8. Trojan

A Trojan can be installed on a computer through phishing or a local device. The purposes of a Trojan can vary, but often this malware hides its files under well-known directories, such as the user’s documents, under the name of a trusted program, such as a web browser. Trojans are commonly used as a backdoor device to collect information from the infected computer. A keylogger is a type of data collection Trojan that can operate over a network or locally through a universal serial bus (USB) as an insider threat attack vector [60,61,62].

Additionally, hardware Trojan attacks refer to malicious modifications of electronic hardware at various stages of its operation. These attacks are a serious security concern for the electronics industry as they can lead to control interference and the leaking of secret data. The growing global demand for electronics makes it a larger point of vulnerability. It requires the adversary to have physical access to the integrated circuits [63,64].

## 4. Processes for Building Cyber Defenses

**Figure 6:**
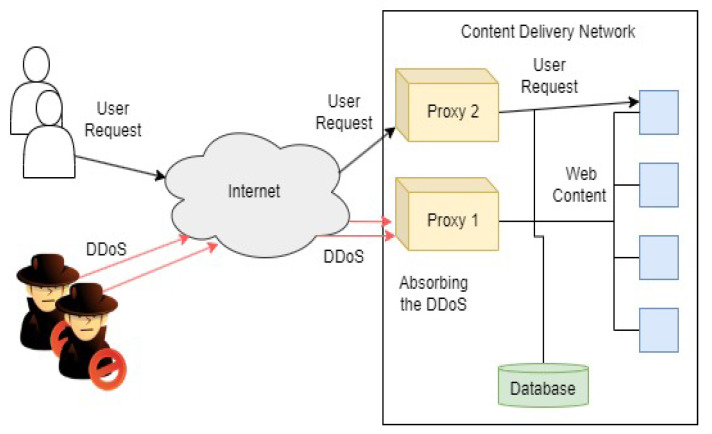
A proxy for protection against network and transport layer DDoS attacks.

**Figure 7:**
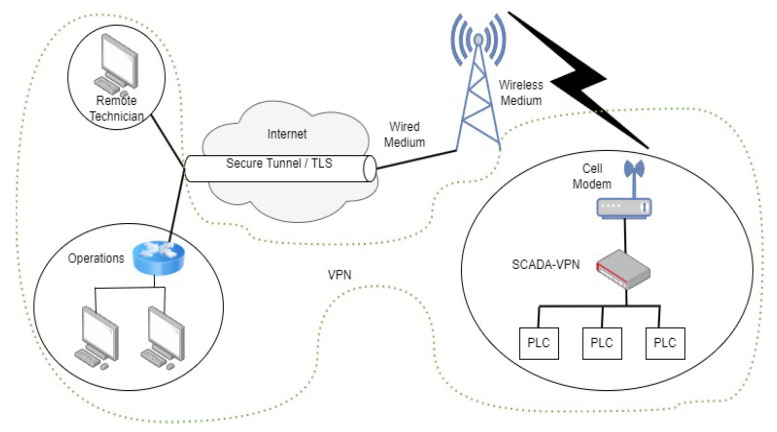
Securing communications (against MITM and FDIA) for SCADA with a VPN.

A successful cyber attack resulting in the unavailability of a critical infrastructure (CI), such as power delivery, can have an economic impact that extends beyond the systems sustaining direct and physical damage. The effects can impact regional and global economies. To identify security risks, analysis is based on what assets are valuable, who wants to attack them, and how they can be compromised. Security decisions are based on understanding the potential damage that can be done to these assets. Recommended cybersecurity for enterprise systems is provided by NIST. Recommended cybersecurity practices for control systems are provided by various organizations, including the Department of Homeland Security (DHS), the North American Electric Reliability Corporation Critical Infrastructure Protection (NERC CIP) standards, and the National Institute of Standards and Technology (NIST) [55]. Two of the common IT technologies to mitigate cyber attacks against networks are illustrated in Figure 6 and Figure 7. The protection scheme for web services and the distribution of content uses load-balancing proxy servers with regionally specific deployments. These proxies are used to absorb DDoS attacks while secondary proxies continue to serve legitimate user requests, as shown in Figure 6. In remotely accessed CI devices, a virtual private network (VPN) can isolate a CI network from the greater internet while leveraging the internet for communication routing, as depicted in Figure 7.

### 4.1. Reason to Train Personnel

Surveys of CI sectors have shown an increased vulnerability to cyber attacks as advances in information technology (IT) are implemented in these sectors. Furthermore, results show that the lack of common knowledge of cybersecurity among personnel is prevalent. Finding personnel lacking knowledge emphasizes the need to increase training in cybersecurity practices for CI personnel. With cyber threat-aware personnel, CIs can be hardened against cyber attacks. The emergence of the IoT in ICS has led to new security challenges, which will require newly developed expertise to prepare for a wide variety of attacks that may emerge from the integration of these systems [65,66,67,68,69].

### 4.2. Threat Matrix and Protection Development Process

**Figure 8:**
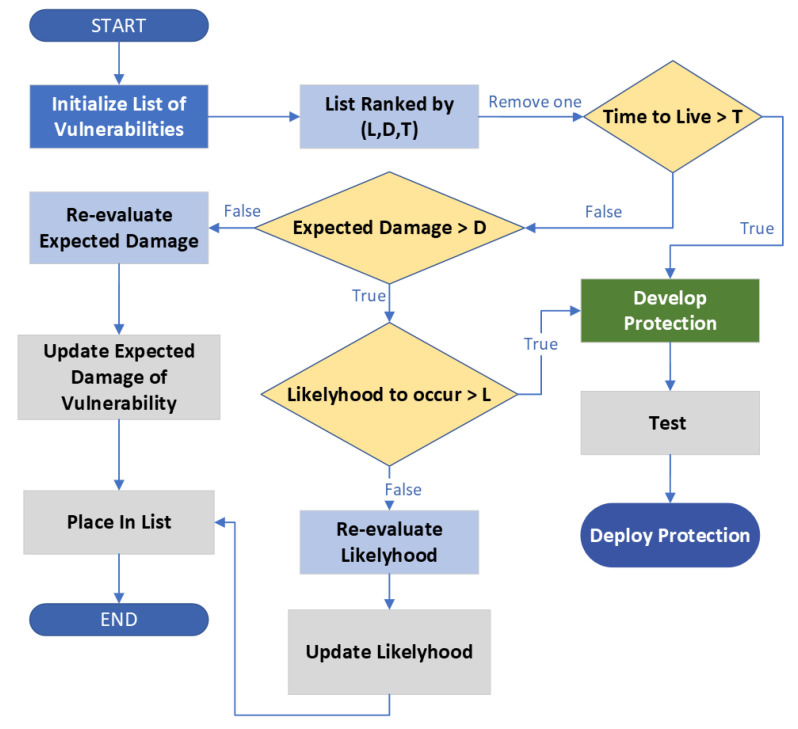
Cybersecurity development process for protecting IT (iterated at minimum acceptable cadence).

Historical cyber attacks have been cataloged and studied through the efforts of Mitre.org, where they have generated an attack matrix for enterprise systems. This matrix provides the sequential stages of a cyber attack from reconnaissance, resource development, initial access, execution, persistence, privilege escalation, defense evasion, credential access, discovery, lateral movement, collection, command and control, exfiltration, and impact. The matrix is organized by techniques and the procedures to execute them. The matrix for ICS is shown in Table 2. For example, one technique is spear phishing, which can involve attaching an (xlsx) file to an email, which side-loads malware into the computer once opened. For each tactic, many procedures may be used depending on the attacker. Mitre and other organizations collaborate in the gathering of procedures under each tactic, and cybersecurity teams around the world can use this matrix to map out their vulnerabilities and areas in which they should develop defenses [70]. The approach to covering an organization’s cyber vulnerabilities can involve an iterative defense development process, as summarized in Figure 8. In this process, the list of vulnerabilities is ranked according to the security operating center (SOC). The time to live of a particular vulnerability is the maximum amount of time that a vulnerability can be ignored. The values for *L*, *D*, and *T* are threshold values for the decisions, also set by the SOC. Such a process can leverage the MITRE matrix to have a comprehensive set of knowledge on attack techniques, with a security team analyzing the priority of vulnerabilities for their organization and iteratively developing defenses based on priority.

### 4.3. Actions of a Defending Organization during a Cyber Attack

Cyber defenders at an organization run through several phases to protect their systems. Initially, a risk assessment phase identifies vulnerabilities. In a subsequent protection phase, the organization develops hardware and software measures to achieve its security goals. In an attack on normal operations, a detection phase will begin in which monitoring mechanisms along with intrusion detection system(s) (IDS) classify abnormal and legitimate network behaviors. Normal and malicious network traffic inside the system are detected. Certain systems will be isolated as they are identified as the root of the cyber attack. The attack will be survived, and in the case of incapacitated OT and IT computer systems, those systems will be brought back into operation as quickly as possible with new security precautions.

Numerous CI sectors are facing challenges in identifying the highest-risk new threats and vulnerabilities. Due to the increased volume and sophistication of cyber attacks, it is crucial to allocate resources strategically and prioritize stopping the most dangerous and likely attacks first [71]. The risk to CI is escalating as the shift from isolated environments to “systems-of-systems” that integrate large information and communications infrastructures continues. The SOC has the responsibility of short-term and long-term planning for the IT/OT future. Guidelines for a cybersecure smart grid system are outlined in [19], emphasizing the enforcement of access control and authentication for all communication throughout the system.

- Every node in the network must have lightweight cryptographic functions.
- Attack detection and mitigating actions are necessary and must be used throughout the smart grid.
- Cyber-security testbeds must be created for the purpose of investigating vulnerabilities in the infrastructure [72].
- The security of network protocols must be designed from the application layer to the MAC layer [19].

### 4.4. IT/OT Practices to Mitigate Malware

A defense strategy against computer malware employs detection and removal. Either signature-based or anomaly-based detection can be used. Furthermore, patching systems to the latest security needs and anti-virus software are other methods used to prevent worms [57,58,73]. A specialized framework for handling computer log-generated data from honeypots, IDS, etc., is proposed for data ingestion, contextualization, and decision-making in formulating an effective and timely response to cyber attacks [74,75]. Approaches to detecting malware are listed below [76]:

- Statistics: An algorithm that utilizes statistical analysis of sample characteristics to determine if the sample is malware.
- Blacklist: The system uses a list of malicious domain names or IP addresses known to be used by ransomware families to identify malware.
- Rule Driven System: A rule-based decision model that finds malware. Rules may include scores such as perceived threats, various threshold values, or rules compatible with malware detection engines.
- Machine Learning-based: Through the use of machine learning (ML) models created with a variety of analysis features, the system can identify malware. ML approaches may analyze instruction opcodes, application programming interface (API) calls, and dynamically linked libraries (DLLs) to build ML classifiers. Systems for detecting malware can identify patterns in the behavior of the malware program.

Active network monitoring using computer network traffic collection tools is an active approach to the detection of security incidents. The network monitoring tools include functionalities for network data collection, parsing of data, the combination of sources into a single data stream, detection of anomalous events in the stream, further exploration of data, and automatic action on the network. Traffic statistics of packet transmission can be analyzed using the Wireshark tool. Collections of packets can be exported by the tool for future analysis. The Elastic Stack distributed database technology with data analytics tools is another option for parsing network activity [77].

IDSs analyze packets or packet flows to detect intrusion into the network by an adversary. The detection method can be signature-based, anomaly-based, or a hybrid detection approach. Intrusion detection systems can also be deployed as a centralized architecture, decentralized, or distributed [78]. It has been found that many existing IDS signatures are based on obsolete attack classes that do not map to modern attacks. Antonia et al. [78] identify and describe the behavior of modern attacks that are not mapped in the IDS attack classes.

### 4.5. The Role of Attribution in Holding Attackers Accountable and Methods to Attribute Attack Network Traffic

The Department of Defense of the U.S. has created techniques for tracing the origins of a cyber attack through intermediaries to the source. The work is presented as a set of techniques for network analyses to attribute a cyber incident to an original perpetrator [79]. Attribution is also discussed in [80]; the authors explain the legal problem that states often avoid penalties for being hosts of cybercrime due to the limited abilities of victims to attribute the attack to the perpetrating state. The paper also suggests that a legal system, specifically an international tribunal, is a more suitable approach to handling attribution, as compared to a technological approach to this aspect of cybercrime. Attribution is a principal aspect of research and spans from the research domains of computer science to international law [81,82,83]. The attribution techniques that can be used are:

- Logging and trace-back queries: Routers may log messages passing through their networks. Backward requests can go up a chain of routers and check if they have seen a previously seen message. As a result, messages that had not previously been classified as harmful can now be attributed. This requires that logging routers be placed in advance, which can lead to cost overruns, poor performance, and numerous other issues. Implementations can also give rise to privacy concerns.
- Input debugging: Defenders can provide an attack pattern as a query to nearby routers, and the router can then report any instances of noticing the pattern. Currently, some distributed denial-of-service (DDoS) attacks are defended against using this strategy. However, it is mostly reactive and only effective against attacks that continually stream data.
- Transmitted message modification: As communications are transferred, routers label them so that their path may be traced. This might affect network performance, increase bandwidth, or interfere with various authentication methods.
- Transmit separate messages: When routing a message, routers also transmit a different message to help with attribution.
- Reconfigure and observe network: Reconfigure the network and go back to a previous phase using the knowledge of what (if anything) changed. Large networks may find it challenging to implement this, and it could lead to new security flaws. On networks owned by others, “controlled flooding” is permitted, but it should only be utilized in specific situations because it could be seen as an attack on third parties.
- Host monitor functions:If a host does not already offer this information, querying functionality can be added (similar to “Query Hosts”). Without the owner’s consent, this is known as a “hack back,” and it calls for strong legal oversight. The information may become substantially less reliable if the host is under the control of an attacker, alerting the attacker.
- Match streams (e.g., via headers, content, timing): Determining which input streams correspond to which output streams involves keeping track of the data streams that are entering and leaving a network or host. This can aid in attribution without requiring knowledge of the network’s or host’s internal state. However, matching can be a challenging technical problem, particularly when dealing with delayed attacks and internal encryption.
- Exploit/force attacker self-identification: To identify the attacker, any information they may have sent, whether on purpose or accidentally, can be used. In some cases, the defender may be able to force the attacker to submit this information. However, many of these techniques rely on extremely technical and specialized approaches (such as beacons, web bugs, cookies, and watermarking) that are easily defeated once an attacker becomes aware of them. When this technique succeeds, it can directly reveal the attacker regardless of how well they have concealed themselves.
- Honeypot/Honeynet: As decoy systems, honeypots and honeynets are used by defenders to bait attackers. Zombie traps (compromised and maliciously controlled computers) and honeynets can instantly reveal any zombies attempting to access the network. However, honeypots and honeynets can only attribute attacks that pass through them, necessitating extensive experience in monitoring and analysis.
- Intrusion detection systems: These systems should be positioned as close as possible to potential attackers (instead of near the defended assets). The placements of the IDSs (which should be close to the attackers) will determine how effective this strategy is. IDSs are notorious for producing many false positives and false negatives, so this strategy frequently necessitates intensive monitoring.
- Filtering of Ingress: Messages can be filtered so that specific links only allow them through if they fulfill particular criteria that make attribution easier. The information for attribution is contained in the message itself, which has the advantage of being transparent to users and requiring no extra storage. The technique’s main limitation is that it can only be used to attribute the locations of internal attacks and often only provides a range of potential attribution values, not a specific location or identity. Frequently, there must be several possible routes for a message to take, leading to uncertainties that reduce the technique’s potency. Network ingress filtering mandates that every message entering a network has a source address in an acceptable range for that network entry point. Using the existing transmission control protocol (TCP)/IP infrastructure, network ingress filtering for IP can be developed and scaled incrementally (one network at a time). The implementation of network ingress filtering by virtually all of the network’s entrance points is necessary for a given network to be successful.
- Spoof prevention: Improving the resistance of protocols or their implementation against fabricated information can significantly reduce the need for examining intermediate systems. However, frequently protocols and/or implementations are difficult to modify to achieve this.
- Secure hosts/routers: The aim is to limit the number of trustworthy intermediate systems that an attacker can access. Although perfect security is unrealistic and this does not accomplish attribution, it simply makes the problem easier to address. This is nevertheless necessary for computer security.
- Surveil Attacker: Direct surveillance of potential or known attackers can prevent advanced attacker strategies. However, this requires prior knowledge of the identity of the expected attacker, and some attackers are very challenging to surveil.
- Employ reverse flow: Data being sent back to the attacker should be marked specifically, and intermediate systems should be able to identify these markings. This can be tracked by stepping stones but requires reverse flow detectors and may be prevented by encryption.
- Combine techniques: Combine multiple approaches. Although it will typically cost more to accomplish, this has a higher chance of success than any other strategy. Special attention must be paid when merging strategies because there is limited expertise in doing so.

## 5. Standards That Address Cyber Attacks

The ISO, NIST, and other high-authority organizations create standards for codifying best practices in establishing cyber-secure environments. Some standards apply to data management in specific sectors, such as health records management. By following these standards, the critical infrastructure industry will be able to mitigate the risks of cyber attacks. Table 3 shows the standards organizations and primary standards that involve cybersecurity. The standards serve as frameworks to develop secure networks and can be used as guides and best practice definitions [84]. The standard NISTIR 7628 provides guidelines for smart grid cybersecurity, including wide-area measurement systems. Although both are considered in the engineering of communications systems for CPS [85].

The ISO 27000 family of standards is an exhaustive and evolving set of standards for traditional and new environments, such as cloud computing. Certifications provided by the ISO for the various standards it has put forth create a guarantee for service users that the system they interact with will be secured. The standard ISO 27018 governs PII security in cloud computing. By applying this standard with existing ISO 27000 family standards, an organization can have a layered approach to managing its data in the context of software as a license on-premise, extending to the cloud context of infrastructure as a service, platform as a service, and software as a service. Agreements between ISO certified service providers and their users are guaranteed by contract. The technology services these standards protect include data, applications, runtime, middleware, operating system, virtualization, servers, storage, and networking. When an organization complies with the standards, it protects its IT and gives confidence to its clients that their information will be managed securely [86].

### NIST Guidelines for Smart Grid Cybersecurity

The National Institute of Standards and Technology (NIST) Guidelines for Smart Grid Cybersecurity [87] provide a framework for developing smart grid cybersecurity. The guide includes use cases to identify high-risk assets, threats, and impacts, as well as high-level security requirements, security architecture, privacy assessment, smart grid standards assessment, and conformity assessment. The guide identifies several adversaries to information systems, including nation-states, organized crime, industrial competitors, disgruntled employees, careless or poorly trained employees, hackers, cyber terrorists, and other criminal elements. The guide also distinguishes various forms of critical infrastructure (CI) on the energy CI, including definitions of (1) physical attacks informed by cyber; (2) cyber-attacks enhancing physical attacks; and (3) the use of a cyber system to cause physical harm.

The use of IDS, antivirus software, and cryptography, are combined in a defense-in-depth approach that focuses on securing PII, power systems assets, IT infrastructures, and communications through layered defenses. Many defenses should be combined to cover the many types of cyber attack threats. The defense-in-depth approach places a focus on people, processes, and technologies. The defense-in-depth strategy aims to place barriers at multiple levels for any cyber attack against the CI. The attacker should be delayed, thus helping the CI to make timely corrective actions. Some of the specific infrastructure mentioned in [87] includes cryptography supporting key, privilege, and certificate management deployed on IT communication technologies, as well as intrusion detection and prevention systems. The cyber attacks experienced are DoS, unauthorized vulnerability probes, botnet command and control, data exfiltration, data destruction, potential physical destruction via alteration of critical software/data. The attacker will combine social engineering and malware to continue their access. The largest threat comes from APTs that select a target and plan and execute a cyber attack against that target over a long time period, with the most damaging attacks being very difficult to detect initially.

## 6. Discussion of Results

This paper has reviewed cyber attack techniques and mitigation strategies; however, it is limited by the vast extent of vulnerabilities that cannot be covered within the paper but which are documented by the community in the Common Weakness Enumeration database. The review provides detailed descriptions of top-level categories of cyber attacks to develop an understanding of the scope of the threat and potential damages. It serves as an introductory point for researchers and industry professionals to enhance their knowledge of existing cyber attacks and mitigation strategies. The review identifies standards that certify an organization’s IT as cyber-secured and offers a retrospective of major cyber attacks launched against critical infrastructures globally in the past 20 years. The paper lists tools and strategies for cyber security teams to defend their infrastructure. Phishing techniques are identified as the initial access point in many cyber attacks on CI and phishing detection and prevention should be further researched.

The reports of major cyber attacks on critical infrastructures have been compiled to understand the types of cyber attacks that are executed, the vulnerabilities that exist, and the typical victims and attackers. The standards that guide the development of cybersecure infrastructure for organizations, along with practical approaches, are listed in one location to help mitigate cyber attacks. Moreover, this review projects that over the next five years, there will be over 1100 significant cyber attacks on global critical infrastructures. The projection shows the rapid growth of significant cyber attacks globally. By reviewing several papers, we developed a framework for phishing and remote sabotage in Figure 5; such an approach utilizes phishing for initial access and lateral movement for access to OT, deploying remote attacks through the OT to impact the critical infrastructure. By further analysis of the adversary techniques, we created a flow chart (Figure 8), which is followed by the SOC in order to develop the necessary defenses for the CI information systems by securing their vulnerabilities via the highest priority ranking.

## 7. Conclusions

The increasing damages caused by cyber attacks, along with their estimated rapid increase in the coming years, make it critical to study them and document their origins, effects, the APTs perpetrating them, and the greater cybercrime economy. The use of ransomware is incredibly profitable when successful, and society pays the price for these interruptions across critical CI sectors such as food, energy, water, etc. [16,21,28,29]. With sophisticated phishing attacks targeting the weakest links in an organization, it is difficult for security teams to secure their networks. Emails, text messages, phone calls, and web pages can all be vectors for a phishing attack. After gaining initial access, escalation of a cyber attack against CI can lead to actions of remote sabotage. FDIA is one potential cyber attack against control systems that is being researched. The need to train all personnel within CIs to be vigilant of such attacks is a valuable investment for a utility [43,44,45,46].

The projection provided by this paper is that the number of significant cyber attacks on critical infrastructures will continue to grow exponentially in the next five years, highlighting the need for increased research in attack detection and prevention. The projection estimates over 1,100 significant cyber attacks on critical infrastructure worldwide in the next five years. While best efforts are made to secure systems, those affected by cyber attacks will mostly be due to selective targeting and efforts by state-sponsored cyber attacks. Certain zero-day exploits and socially engineered credential theft are expected to remain perpetual weak points in computer networks for the foreseeable future [5]. Cyber defenses should include training all personnel to be aware of cyber threats. Further, a process for developing protection mechanisms for the IT/OT should be used at the SOC. The ability to attribute a cyber attack to the original attacker is possible through many approaches. A combined approach to attribution is likely to be the most successful in identifying the original attacker and allows the CI to cooperate with law enforcement. With the dynamic and computerized nature of the smart grid, there are now more pressing cybersecurity requirements on the energy CI than ever before. 

## Figures and Tables

**Table 1:** Abridged list of significant cyber attacks in recent years.

Adversary Technique	Types of Cyber Attacks Used	CI	Impact on CI Operations
Initial Access, Execution, Lateral Movement, Impact	Phishing, Remote Desktop, BlackEnergy3 (FDIA)	Energy (2015–2016)	Opened breakers in substations in Ukraine, causing 230,000 customers to lose power.
Initial Access, Execution, Impact	Ransomware	Energy (2021)	Fuel shortages for Southeast US with gas prices rising (9–16 cents per gallon) and 10,600 stations without gas. The Colonial Pipeline billing system shutdown for six days.
Initial Access, Execution, Impact	Stuxnet Worm, Zero-day Vulnerabilities	IT (2009–2011)	Shutdown of uranium enrichment facilities in Natanz, Iran.
Initial Access, Execution, Persistence, Collection	Trojan Laziok, reconnaissance malware	Energy (2014)	Gathered information from devices on the network that has vulnerabilities.
Initial Access, Execution, Collection, Impact	Ransomware	Food (2021)	Data of customers, suppliers, and employees were stolen. Productivity was reduced, access to some systems was blocked. A USD 11 million ransom was paid. Operation servers were shutdown and operations halted.
Initial Access, Execution, Impact	Ransomware	Healthcare (2021)	All Hospital appointments and radiology services were impacted, the ransomware affected Windows operating systems. The failure was experienced across national networks.
Initial Access, Execution, Impact	Phishing and Ransomware (Roobinhood)	Financial (2019)	Trading services of exchange halted and maintained offline, as computer systems were maliciously encrypted.
Initial Access, Execution, Collection	Phishing	Financial (Disclosed 2017)	The Equifax data breach resulted in the theft of personal data belonging to 140 million Americans and caused the company’s share price to drop by 13%.
Initial Access, Execution, Persistence, Collection	Trojan Malware	Financial (2016)	The malware recorded debit cards and their pins from compromised ATM machines. Approximately USD 194,000 was stolen.
Initial Access, Execution, Collection, Impact	WannaCry Ransomware Cryptoworm	Energy (2017)	Worldwide Microsoft Windows operating systems were ransomed using an older Windows systems vulnerability EternalBlue.
Initial Access, Execution, Collection	Phishing, Ransomware	IT (2021)	The Accellion data breach and ransomware attack led to the theft of data in the Accellion data management service.
Initial Access, Execution, Lateral Movement, Impair Process Control	Supply Chain Ransomware	IT (2021)	Over 1500 businesses and organizations halted operations. A software updater released by an IT company, operating as a managed service provider
Initial Access, Execution, Inhibit Response Function, Impact	Ransomware	Municipal Services (2018)	Required over 5000 government computers to be shut down for 5 days to resolve the attack. Affected servers that were used to issue police warrants and employ new hiring processes, as well as official city complaints could not be submitted.

**Table 2:** Complete MITRE ATTACK Matrix for ICS—top level techniques as of November 2022 [70].

Initial Access	Execution	Persistence	Privilege Escalation	Evasion	Discovery	Lateral Movement	Collection	Command and Control	Inhibit Response Function	Impair Process Control	Impact
Drive-by Compromise	Change Operating Mode	Hardcoded Credentials	Exploitation for Privilege Escalation	Change Operating Mode	Network Connection Enumeration	Default Credentials	Adversary-in-the-Middle	Commonly Used Port	Activate Firmware Update Mode	Brute Force I/O	Damage to Property
Exploit Public-Facing Application	Command-Line Interface	Modify Program	Hooking	Exploitation for Evasion	Network Sniffing	Exploitation of Remote Services	Automated Collection	Connection Proxy	Alarm Suppression	Modify Parameter	Denial of Control
Exploitation of Remote Services	Execution through API	Module Firmware		Indicator Removal on Host	Remote System Discovery	Hardcoded Credentials	Data from Information Repositories	Standard Application Layer Protocol	Block Command Message	Module Firmware	Denial of View
External Remote Services	Graphical User Interface	Project File Infection		Masquerading	Remote System Information Discovery	Lateral Tool Transfer	Detect Operating Mode		Block Reporting Message	Spoof Reporting Message	Loss of Availability
Internet Accessible Device	Hooking	System Firmware		Rootkit	Wireless Sniffing	Program Download	I/O Image		Block Serial COM	Unauthorized Command Message	Loss of Control
Remote Services	Modify Controller Tasking	Valid Accounts		Spoof Reporting Message		Remote Services	Monitor Process State		Data Destruction		Loss of Productivity and Revenue
Replication Through Removable Media	Native API					Valid Accounts	Point & Tag Identification		Denial of Service		Loss of Protection
Rogue Master	Scripting						Program Upload		Device Restart/Shutdown		Loss of Safety
Spearphishing Attachment	User Execution						Screen Capture		Manipulate I/O Image		Loss of View
Supply Chain Compromise							Wireless Sniffing		Modify Alarm Settings		Manipulation of Control
Transient Cyber Asset									Rootkit		Manipulation of View
Wireless Compromise									Service Stop		Theft of Operational Information
									System Firmware		

**Table 3:** Standards that govern the frameworks, best practices, and specifications for cybersecure information systems.

Body	Standard	Core Contribution	Adversary Technique Mitigated
ISO/IEC	27018:2019	Provides security techniques and a code of practice for the protection of personally identifiable information in public clouds with guidelines based on ISO/IEC 27002.	Initial access, discovery, collection
ISO/IEC	27037:2012	Secure techniques for identifying, collecting, and preservation of digital evidence, and will assist organizations in attributing blame based on digital evidence.	All Techniques
ISO/IEC	27040:2015	Guidelines on the creation of a low-risk data management security system. This includes security for devices and media, applications, and services, and security relevant to end-users.	Initial access, lateral movement, inhibit response function, privilege escalation
ISO	22301	A framework for organizations to be resilient and continue business operations during and after a cyber attack. Develops business continuation plans in the event of a disruption.	Inhibit response function, impair process control, impact
ISO/IEC	27001	A framework for the implementation of secure corporate enterprise computer systems. Details of the implementation of security controls to manage risks.	All Techniques
ISO/IEC	27002	Extends ISO/IEC 27001 standard with guidelines on best practices. Provides organizations with generic information security controls, including implementation guidance. Defined use for an information security management system based on ISO/IEC 27001.	All techniques
ISO/IEC	27031	Information and communication technology guidelines for business continuity. Covering concepts and best practices.	-
ISO/IEC	27032	Guidance on cybersecurity management system, and best practices for information security.	Lateral movement, inhibit response function, collection
ISO/IEC	27701	Specifications for building a privacy information management system that is based on ISO 27001. Published to address a growing need for a framework for global data privacy.	Collection
NIST	Cyber-security framework	Guideline for managing cybersecurity risks based on the existing best practices, guidelines, and standards provided in three components: core, implementation tiers, and profiles.	All techniques

## Data Availability

Not applicable.

## Abbreviations

SCADA	Supervisory Control and Data Acquisition
DoS	Denial of Service
CIA	Confidentiality, Integrity, and Authenticity
DNP3	Distributed Network Protocol 3
CI	Critical Infrastructure
IT	Information Technology
OT	Operational Technology
RAAC	Ransomware-as-a-Corporation
CSIS	Center for Strategic and International Studies
VPN	Virtual Private Network
MITM	Man-in-the-Middle
DNS	Domain Name System
TCP	Transmission Control Protocol
SSL	Secure Socket Layer
FDIA	False Data Injection Attack
ICMP	Internet Control Message Protocol
CWE	Common Weakness Enumeration
ARP	Access Resolution Protocol
SOC	Security Operating Center
APT	Advanced Persistent Threat
